# Setting priorities in health research using the model proposed by the World Health Organization: development of a quantitative methodology using tuberculosis in South Africa as a worked example

**DOI:** 10.1186/s12961-016-0081-8

**Published:** 2016-02-09

**Authors:** Damian Hacking, Susan Cleary

**Affiliations:** Health Economics Unit, Faculty of Health Sciences, University of Cape Town, Observatory, Cape Town, South Africa

**Keywords:** Health research, Priority setting, Quantitative model, Tuberculosis, WHO

## Abstract

**Background:**

Setting priorities is important in health research given the limited resources available for research. Various guidelines exist to assist in the priority setting process; however, priority setting still faces significant challenges such as the clear ranking of identified priorities. The World Health Organization (WHO) proposed a Disability Adjusted Life Year (DALY)-based model to rank priorities by research area (basic, health systems and biomedical) by dividing the DALYs into ‘unavertable with existing interventions’, ‘avertable with improved efficiency’ and ‘avertable with existing but non-cost-effective interventions’, respectively. However, the model has conceptual flaws and no clear methodology for its construction. Therefore, the aim of this paper was to amend the model to address these flaws, and develop a clear methodology by using tuberculosis in South Africa as a worked example.

**Methods:**

An amended model was constructed to represent total DALYs as the product of DALYs per person and absolute burden of disease. These figures were calculated for all countries from WHO datasets. The lowest figures achieved by any country were assumed to represent ‘unavertable with existing interventions’ if extrapolated to South Africa. The ratio of ‘cost per patient treated’ (adjusted for purchasing power and outcome weighted) between South Africa and the best country was used to calculate the ‘avertable with improved efficiency section’. Finally, ‘avertable with existing but non-cost-effective interventions’ was calculated using Disease Control Priorities Project efficacy data, and the ratio between the best intervention and South Africa’s current intervention, irrespective of cost.

**Results:**

The amended model shows that South Africa has a tuberculosis burden of 1,009,837.3 DALYs; 0.009% of DALYs are unavertable with existing interventions and 96.3% of DALYs could be averted with improvements in efficiency. Of the remaining DALYs, a further 56.9% could be averted with existing but non-cost-effective interventions.

**Conclusions:**

The amended model was successfully constructed using limited data sources. The generalizability of the data used is the main limitation of the model. More complex formulas are required to deal with such potential confounding variables; however, the results act as starting point for development of a more robust model.

**Electronic supplementary material:**

The online version of this article (doi:10.1186/s12961-016-0081-8) contains supplementary material, which is available to authorized users.

## Background

The demand for health research far outstrips the current financial and capacity resources made available to do so [1], and hence it is important to set priorities when making decisions about what research to undertake. Furthermore, priorities should be set in a transparent, rational and systematic manner. This need was articulated by the Commission on Health Research for Development in 1990 [2], and led to the formation of the Council on Health Research for Development (COHRED), which celebrates its 20th anniversary this year. COHRED is arguably the world leader in priority setting in health research, and over the past two decades has fostered an international movement towards procedural priority setting. While COHRED does not endorse a specific priority setting process, they do provide general guidelines on best practices for countries that wish to set health research priorities [3]. Broadly, processes should involve quantitative data inputs as well as participation from stakeholders, such as researchers, funders, politicians, community members, health workers, economists and civil servants. These two inputs are then used to identify priorities, which can range from specific research questions to broader disease areas. The identified priorities are subsequently ranked in order of importance, usually with a greater focus on stakeholder input and less focus on quantitative data inputs. There are a variety of frameworks, like the Combined Approach Matrix [4] or the Essential National Health Research [5] framework, which provide guidelines on priority identification and ranking, such as what data is relevant, which stakeholders should be involved in the identification of research priorities, what criteria are used in priority identification, and how the research priorities identified are ranked. However, despite the COHRED guidelines and a plethora of frameworks, priority setting still faces significant challenges, such as the uncertainty of health research outcomes, ensuring that the priority setting process is transparent and fair, and defining relevant criteria to identify and rank priorities [6]. This paper will address the challenge of subjectivity in ranking criteria, and the need for a clearly defined, transparent and quantitative methodology to rank priorities [7]. Importantly, this methodology must also be relatively simple, as priority setting can be most beneficial in resource-scarce developing countries, which generally have a paucity of data and expertise relevant for ranking priorities [7]. The model presented in this paper does not intend to replace the holistic and inclusive approach advocated by COHRED, but rather is presented as a resource to supplement the priority setting process.

**Fig. 1:**
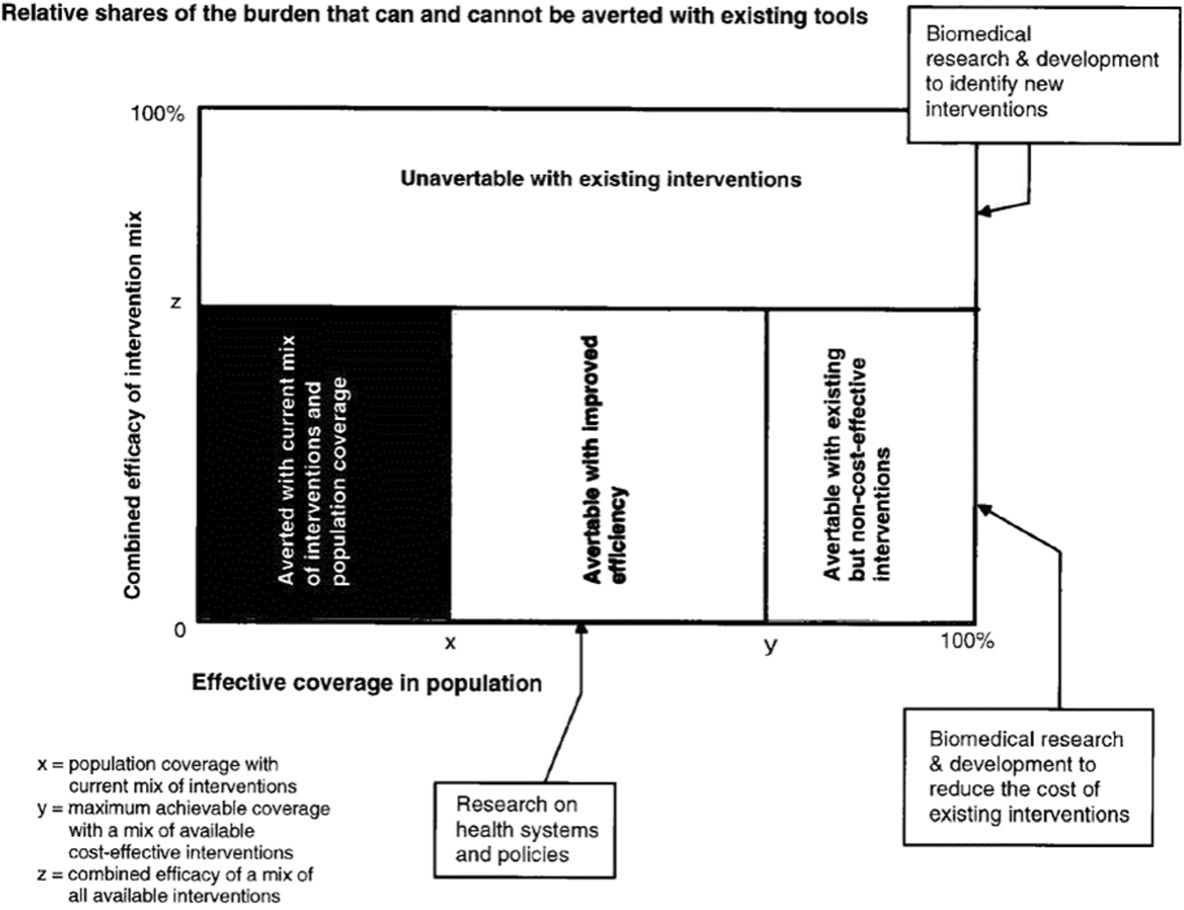
WHO model for ranking of research priorities [8]. Ability to tackle the burden of disease is represented by the efficacy of interventions, and the coverage in the population. Improvements in coverage can be gained via either health systems research or biomedical research to reduce the cost of interventions. Efficacy gains can arise from research to identify new interventions

Current ranking techniques are divided into two categories, either direct or indirect [3]. Direct techniques involve contrasting two priorities and selecting the one of greater importance such as a discrete choice method. Indirect techniques utilise a scoring system that scores a priority based on a list of criteria and then calculates a cumulative score using a formula that incorporates all the criteria listed. While this latter method increases the transparency and standardises ranking of priorities, the criteria used, and their weighting, is determined by stakeholder input and is therefore highly subjective. The World Health Organization (WHO) proposed an alternative, quantitative-based, model for ranking priorities by research area in 1996, as outlined in Fig. 1.

In this model, Disability Adjusted Life Years (DALYs) are used to construct a quadrangle for a particular disease. This quadrangle consists of two axes, namely how much of the population affected could be treated with interventions, and how effective the interventions could be at treating the disease. Four subdivisions are then listed. Firstly, how well the current interventions are working, both in terms of treatment efficacy and population coverage, defined as the ‘averted with current mix of interventions and population coverage’ section. Secondly, what additional coverage could be obtained with gains in health systems and policy research, defined as the ‘avertable with improved efficiency’ section. Thirdly, what gains in coverage could be obtained with biomedical research to reduce the cost of interventions, defined as the ‘avertable with existing but non-cost-effective interventions’ section. Finally, gains in effectiveness of interventions from basic and clinical research to identify new interventions, defined as the ‘unavertable with existing interventions’ section. By using this model, priority areas can be identified and ranked in terms of disease and research area automatically, as the area of a section would indicate the degree to which research in that disease field (e.g. HIV health systems and policy research) should be a priority relative to other disease fields. However, in its current state, the WHO model has some major flaws, both in the methodology and inherent to its structure.

Firstly, one of the main assumptions of the WHO model is that the sum of all the components contributing to coverage and efficacy of interventions would neatly sum to 100% coverage and 100% efficacy. By way of example, in Fig. 1, current interventions (x value on x-axis) cover approximately 30% of the population, with an additional 40% coverage possible with improvements in efficiency, and 30% additional coverage achievable with use of existing but non-cost-effective interventions. However, it is possible to envisage a situation where just improving efficiency could increase coverage by an extra 60%, or just using non-cost-effective interventions could increase coverage by an extra 60%. The total coverage in such a case would thus be the baseline of 30% plus 120% if both efficiency improvements were made and non-cost-effective interventions adopted, to a total of 150% coverage. As it is not possible to achieve such a thing, it is important to adapt the model to show a relativistic improvement, as the problem arises due to the assumption of mutual exclusivity in the model. The model assumes that, with the current burden of disease, you could increase coverage by either increasing efficiency or using non-cost-effective interventions. However, these gains are both based on the current disease burden, yet if improvements in efficiency were to be made, then the disease burden would change and the contribution that non-cost-effective measures could make would only apply to the new disease burden. Thus, it makes more sense to look at relativistic changes, such as the subsequent impact of the adoption of non-cost-effective interventions if improvements to efficiency were made, or vice versa.

Secondly, the current construction of the WHO model assumes that use of non-cost-effective interventions and increases in health system efficiency will only increase population coverage, and not the efficacy of the interventions. However, there is no reason to assume that this will always be the case, and therefore the model would also need to be amended to allow for changes in both population coverage and efficacy from gains in health systems efficiency and use of non-cost-effective interventions. Finally, the current axes are presented in terms of percentage coverage and percentage efficacy of interventions against a disease. However, because disease burdens vary, it limits the viability of cross-comparisons between diseases. Furthermore, it is unclear what ‘coverage in the population’ means, as it could apply to both provision of treatment for those with the disease as well as preventive measures for the ‘at risk’ population. Because of these limitations, the axes of the model should also be adapted to present the results in terms of absolute disease burden figures, and not percentage coverage or efficacy. Furthermore, the y-axis of ‘coverage in the population’ must be more clearly defined and able to incorporate both curative and preventive measures.

It is perhaps for these reasons that the WHO model has largely remained unused, although it has been referenced as a model that could be incorporated into current priority setting frameworks, such as the Essential National Health Research [5] or Combined Approach Matrix frameworks [4]. This is compounded by the fact that, even if the model were made theoretically sound, practically, it is not an easy model to construct since data on treatment coverage and efficacy are not widely available and no formal methodology has ever been proposed for the construction of the quadrangle. Therefore, the present paper focuses on developing an amended model based on the principles of the WHO model and attempts to define an explicit methodology for its construction using quantitative data. The viability of the amended model was then explored using tuberculosis (TB) in South Africa as a worked example.

## Methods

### Amendment of the WHO model

**Fig. 2:**
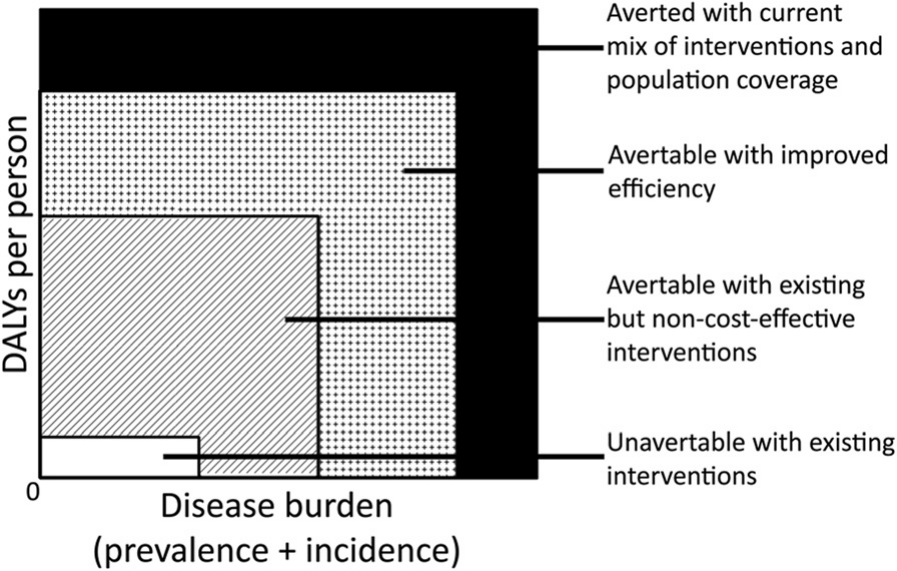
Amended model based on the original WHO model. DALYs are represented as a product of disease burden (incorporating both incidence and prevalence measures) and DALYs due to the disease per person. DALYs which are currently being averted are appended to the ends of the axes, while those that currently exist could be averted with either health systems research to improve efficiency, biomedical research to reduce the cost of existing interventions, or research on new interventions to tackle the portion of the burden that not even the best existing interventions can avert

The axes of the amended model were changed to those of disease severity (as measured by DALYs per person) and disease burden (as measured by both prevalence and incidence). The revised model thus seeks to reduce severity and disease burden, and subsequently the size of the quadrangle, as opposed to increasing coverage and efficacy as in the original WHO model. Similar to the WHO model, the amended model acknowledges that there is a particular severity and burden of the disease that is unavertable with existing interventions, as well as a larger severity and burden that the current intervention mix is averting. In addition, the portion of the disease that is avertable, could be averted either via increases in efficiency of the health system or use of existing but non-cost-effective interventions, as stipulated by the WHO model. In the amended model, these two routes are allowed to have impact on both burden and severity measures of the disease (Fig. 2).

The amended model also nests the ‘avertable with existing but non-cost-effective interventions’ section within the ‘avertable with improved efficiency’ section (and vice versa, if so desired), to address the issues of relativistic changes and mutual exclusivity in the original WHO model.

Finally, the revised model appends the additional disease burden and DALYs that are ‘averted with current mix of interventions and population coverage’ to the outer limits of each axis.

### Application of amended model to TB

**Fig. 3:**
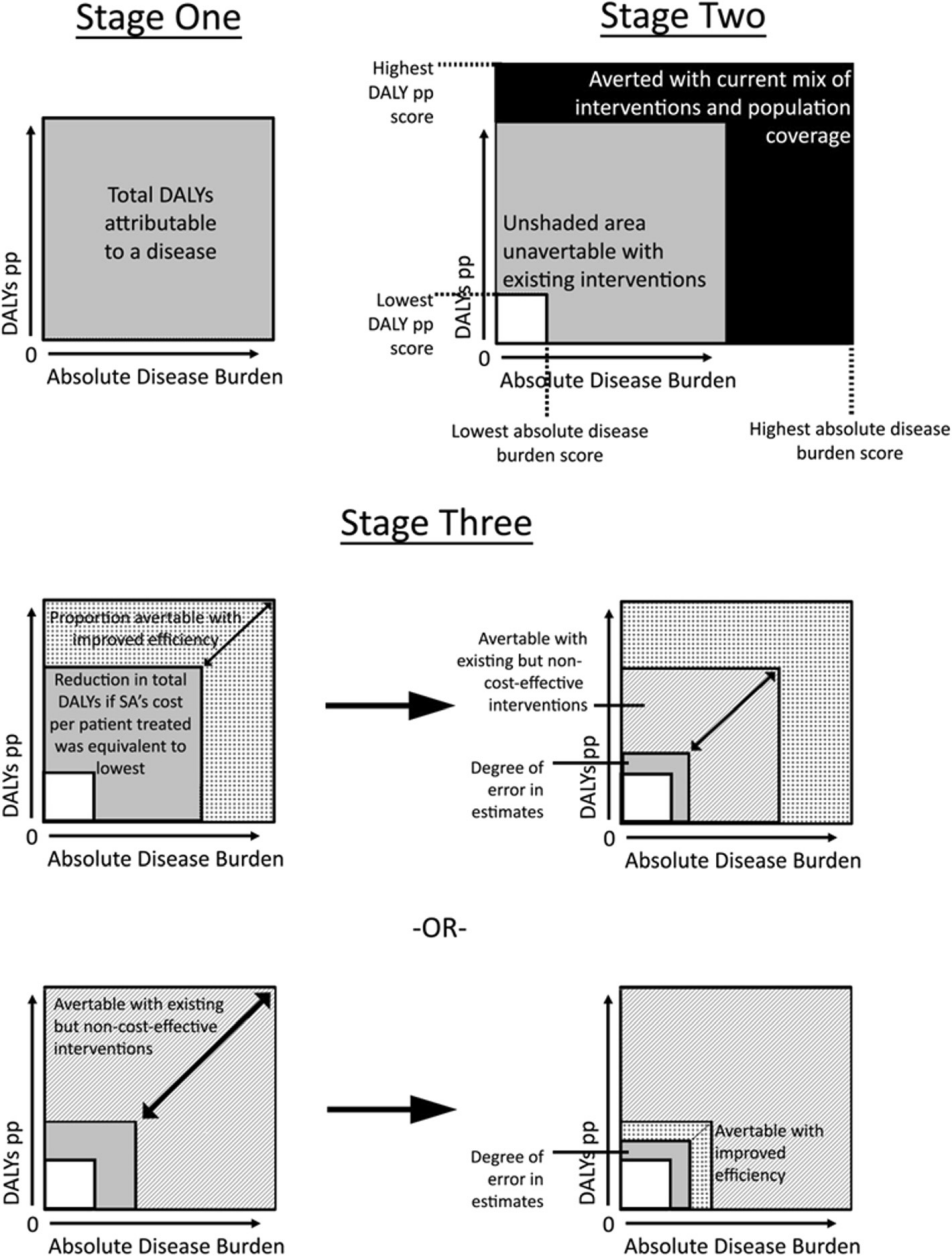
Construction of amended model. Stage 1: Construction of the two axes to create the total DALYs attributable to a particular disease. Stage 2: Determination of the DALYs currently being averted as well as those unavertable with existing interventions, based on the best and worst performing member states. Stage 3: Division of current DALYs into those that could be reduced with improvements in health systems, and those that could be reduced with reduction in costs of existing interventions

The construction of the amended model for TB involved three stages, as outlined in Fig. 3. To construct the x-axis, both incidence and prevalence estimates were consulted. The Global Tuberculosis Control report [9] provides prevalence measures as point estimates of the number of individuals with a disease in the population at a given time based on survey data. It also provides incidence measures as estimates of new and recurring cases of TB over a 1 year period based on modelling. Disease burden was calculated as the sum of the incidence and prevalence per 100,000 estimates for South Africa. While the simple addition of prevalence and incidence estimates may result in overestimation of the disease burden (as some of the incident cases will be measured twice by the point prevalence), it does allow for a more accurate reflection of diseases with high incidence but low prevalence (e.g. diarrheal diseases), as well as chronic diseases that may have a low incidence, but a much higher prevalence (e.g. hypertension). It also allows for both preventive measures, which largely impact disease incidence, and curative measures, which impact disease prevalence, to be accounted for. Therefore, it was important to include both measures of incidence and prevalence in the calculation of disease burden. The disease burden was then applied to South Africa’s population size in 2004 to get an absolute disease burden score using equation 1:

1 $$ \begin{array}{l}\left( prevalence+ incidence\; per\;100,000\right)\ \mathrm{x}\ \left( South\  African\; population/100,000\right)\\ {}\kern10em = absolute\; disease\; burden\end{array} $$

For the y-axis, South Africa’s DALYs per person (pp) were calculated using equation 2, with age standardised DALY rates sourced from WHO 2004 disease burden data [8] and TB prevalence and incidence rates from 2004 in the 2006 Global Tuberculosis Control report [9].

2 $$ DALYs\; per\;100,000/\left( prevalence+ incidence\; per\;100,000\right)= DALYs\; per\; per son $$

This quadrangle therefore represents the entire DALYs of TB for South Africa, which can be attributed to the three mechanisms outlined in the amended model, namely the proportion which is unavertable with existing interventions, the proportion which is avertable with improved efficiency, and the proportion which is avertable with existing but non-cost-effective interventions.

The second stage was to determine that component of the quadrangle which was currently unavertable with existing interventions, and to append the additional DALYs that are currently being averted. To determine this, DALYs pp and disease burden were calculated for each country where both data was available (Additional file 1). Those countries which had no DALYs, incidence, or prevalence per 100,000 were excluded. Of the remaining countries, the lowest DALY pp score was appended to the y-axis. For the x-axis, the lowest disease burden score was extrapolated using equation 3 to get a hypothetical absolute lowest disease burden for the South African population.

The resultant quadrangle was assumed to estimate that portion of the DALYs unavertable with existing interventions, as these are the best figures that any country has been able to achieve. However, even in the best performing countries used to construct this estimate there are other determinants of health, such as climate. These factors may prevent optimal treatment of a disease (e.g. monsoons) or assist in treatment efficacy (e.g. warm climates). Thus, there is a degree of uncertainty around the estimate.

3 $$ \left( country\; with\; combined\; lowest\; incidence+ prevalence\; per\;100,000\right)\ \mathrm{x}\ \left( South\  African\; population/100,000\right) $$

To determine the degree of disease burden currently being averted, the worst performing countries, both in terms of DALYs pp and disease burden, were identified. The same methodology used to calculate the hypothetical best disease burden for South Africa was used to calculate the hypothetical worst disease burden for South Africa. These figures were then appended to the current DALY pp and disease burden estimates. The premise being that South Africa is averting at least as much as the difference between itself and the worst performing countries, if not more so, as even the worst performing countries are likely to be combating TB in some manner.

Two approaches were taken to subdivide the remaining disease burden (Stage 3 of Fig. 3). In the first approach, the component which could be averted with improved efficiency was first calculated, and then the remaining disease burden was used to calculate what could be averted with existing but non-cost-effective interventions. In the second approach, the component which could be averted with existing but non-cost-effective interventions was first calculated, and then the remaining disease burden was used to calculate what could be averted with improvements in efficiency. For both cases, calculations were performed in the same manner. To determine the proportion avertable with improved efficiency, cost per patient treated (CPP) data from 2008 and 2009 (both in US dollars, and adjusted for purchasing power parity) was used. Data from 2008/2009 was used as this data has only recently been recorded by WHO and hence there was no data available for 2004. This value was weighted by DALY pp data to control for the potential differences in outcomes of the treatment, as such:

4 $$ cost\; per\; patient\; treated/\left[1/ DALY\; pp\right]= weighted\; cost\; per\; patient $$

In equation 4, countries with poor treatment outcomes (i.e. high DALY pp values) will have a small denominator, and hence their cost per patient will be inflated. Alternatively, countries that spend slightly more, but achieve better outcomes, will have a larger denominator and hence their cost per patient will be deflated. The ratio between South Africa’s weighted CPP and the lowest weighted CPP obtained was hypothesized to represent the proportion of total disease burden reduction which could be obtained with improvements in efficiency. It was assumed that the reduction would be an equivalent reduction in DALYs pp and disease burden, and hence the ratio between the axes remained fixed. The degree of the disease which is avertable using existing but non-cost-effective interventions was calculated using TB cost-effectiveness data from the Disease Control Priorities Project report [10]. This report models efficacies of current treatments for TB in six global regions, as well as hypothetical best efficacies of interventions in those regions and their respective costs. The ratio between the highest average efficacy achievable by all the existing interventions mentioned in the sub-Saharan Africa region (irrespective of cost) and the efficacy of South Africa’s current intervention program was assumed to represent the proportion of the disease burden which could be averted with existing but non-cost-effective interventions. Similarly, it was assumed that this would be equally reflected in disease burden and DALY pp reductions, and hence the ratio between the axes remained fixed.

## Results and discussion

### Construction of model

Table 1 summarises all the data used in construction of the amended model. South Africa had a DALY pp value of 1.48. The country with the lowest value was Kiribati, with a value of 0.12, whereas the country with the highest value was the United Arab Emirates, with a value of 13.47 DALYs pp. This is a surprising result for the United Arab Emirates, as dry climates are known to speed TB recovery [11]. Potential explanations for this include the relatively high contribution by emigrants from South and Southeast Asian countries [12], the increase in multiple drug resistant TB, as well as a novel strain of TB among the indigenous population [13]. In terms of disease burden, the United Arab Emirates has a combined prevalence and incidence per 100,000 of 5.7, ranking seventh lowest amongst the member states (Additional file 1).

**Table 1:** List of figures used to construct amended model

Data type	Value	Country
DALY data		
DALYs per person (PP)	1.48	South Africa
Best DALYs PP	0.12	Kiribati
Worst DALYs PP	13.47	United Arab Emirates
Disease burden (DB) data		
DB/100,000	1,683	South Africa
DB	682,322.47	South Africa
Best DB/100,000	1.78	Monaco
Best DB	721.65	South Africa
Worst DB/100,000	3,342	Namibia
Worst DB	1,354,915	South Africa
Cost per patient (CPP)		
Weighted CPP	404.45	South Africa
Best weighted CPP	14.29	Yemen
Fold reduction	28.30	
Weighted CPP PP adjusted	621.33	South Africa
Best weighted CPP PP adjusted	22.81	Namibia
Fold reduction PP adjusted	27.24	
Cost efficiency (DCPP) data		
Sub-Saharan Africa baseline treatment active infection	33%	
Sub-Saharan Africa best treatment active infection	71%	
Sub-Saharan Africa best treatment latent infection	80%	
Fold increase	2.15	

DCPP, Disease Control Priorities Project

**Fig. 4:**
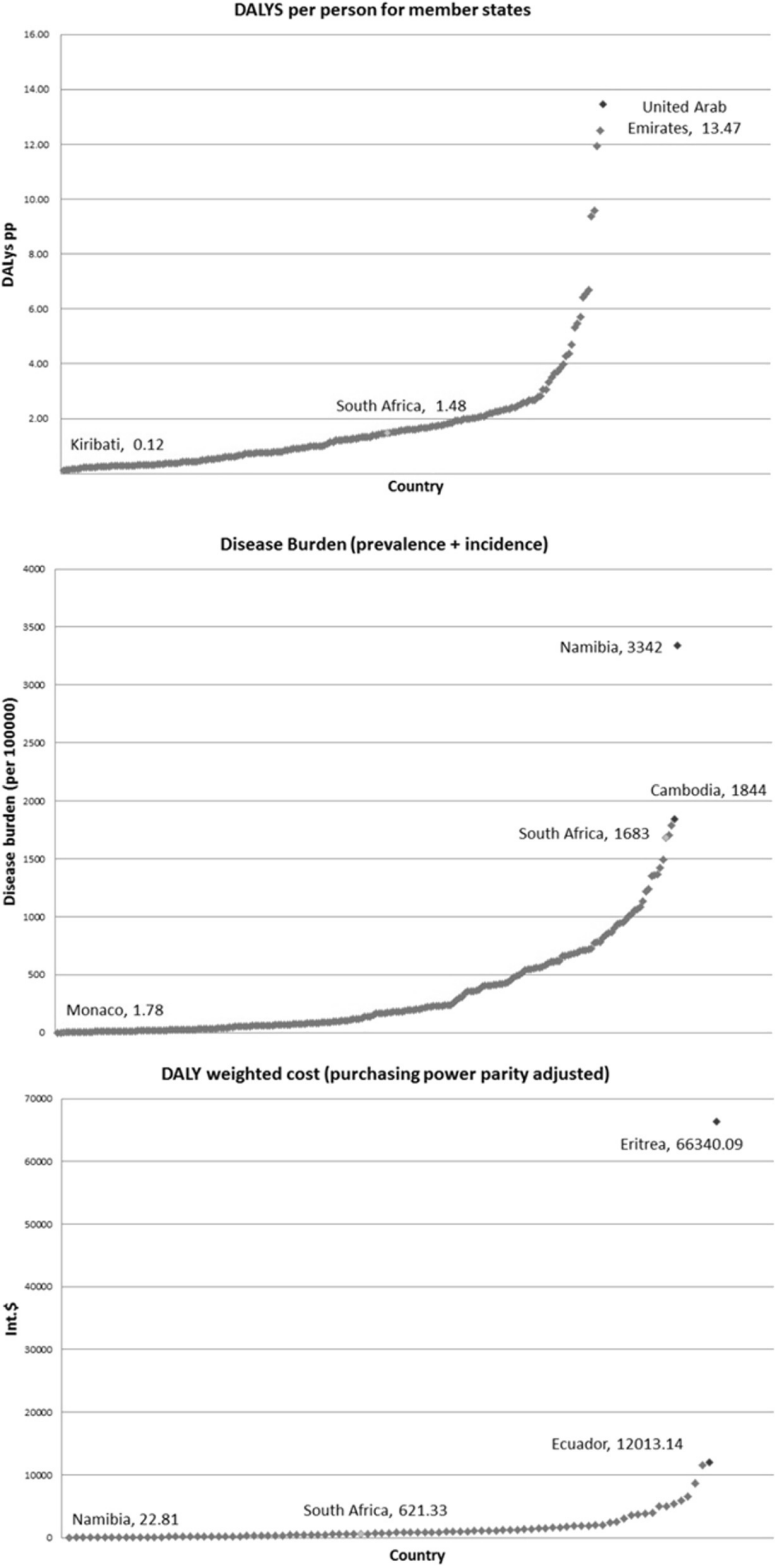
DALYs per person, Disease Burden and DALY weighted cost (purchasing power parity adjusted) for all member states. Lowest scorers are highlighted in green, highest in red, and South Africa in yellow for reference. Namibia and Eritrea are considered outliers in the Disease Burden and DALY weighted cost graphs, respectively, and so the second highest countries are also included

This suggests that relatively few people contract TB, but that it is a particularly severe strain. While this does call into question the validity of using the United Arab Emirates as a baseline for worst case scenario, the Cook Islands, Burkina Faso, Mali and Togo all have DALYs pp of greater than 9 (Additional file 1), and have vastly disparate climates, genetics and disease profiles. Hence, a DALY pp value of 13.47 is within acceptable range, but a value between 9 and 13 could be a more conservative estimate. The lowest DALY pp country, Kiribati, has one of the highest incidence rates of TB in the Western Pacific region [14]; however, it also has stringent treatment guidelines, with patients being quarantined for 2 months during treatment, and then further confined to a specialised centre until fully healed [15]. This combination of high incidence and strict treatment could account for the resultant low DALY pp value. The small population may limit the generalizability of such a treatment, and this is supported by similar small countries also achieving low DALY pp rates (Additional file 1); however, it should be noted that larger countries, like Australia and Switzerland, have DALY pp values of 0.15 and 0.17, respectively. Therefore, both United Arab Emirates and Kiribati figures were used in the construction of the amended model as they did not deviate from the general trend of the member states (Fig. 4).

**Fig. 5:**
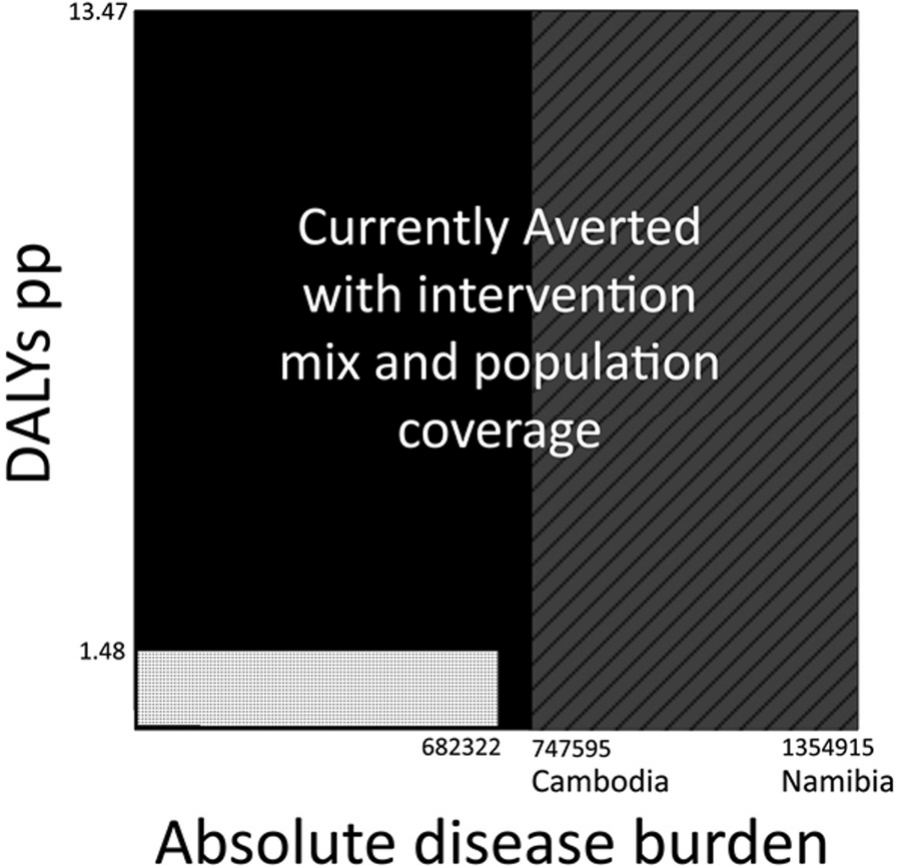
Amended model of TB DALYs for South Africa. South Africa has an average DALY per person (pp) score of 1.48 and absolute disease burden of 682,322. Therefore, 0.12 DALYs pp and a disease burden of 722 are unavertable with existing interventions, but are too small to see at this scale. South Africa is currently averting a DALY pp figure of 13.47 and an absolute disease burden of between 747,595 and 1,354,915

In terms of disease burden, South Africa had a combined absolute incidence and prevalence of 682,322.5. Monaco had the lowest combined prevalence and incidence of 1.78 per 100,000, whereas Namibia had the highest with 3,342 per 100,000. This extrapolated to a hypothetical best and worst disease burden for South Africa of 721.65 and 1,352,915, respectively. Again, the high levels of TB in Namibia, which is a desert area, are unexpected. Possible explanations for this include the high co-morbidity with HIV [16] as well as a high rate of drug resistant TB [17]. Interestingly, similar to the United Arab Emirates, Namibia appears to be plagued by a specific subset of TB which could be exacerbating its disease burden [18]. These differences in disease profiles do limit the generalizability of using Namibia as a worst case scenario, particularly as Namibia is unique in having both the highest incidence per 100,000 and the highest prevalence per 100,000 (by way of example, Cambodia has the second highest prevalence but only the 11th highest incidence). Namibia’s disease burden score is also disproportionately high compared to the other top five countries (Cambodia, Swaziland, Central African Republic and South Africa) which range from 1,683 to 1,844 (Fig. 4). On the other end of the scale, Monaco had the lowest disease burden score; however, this was relatively similar to other low scoring countries. Therefore, Monaco was used in the construction of the amended model, and both Namibia and Cambodia were used due to the potential of Namibia as an outlier. From this data, the amended model was constructed as shown in Fig. 5 (Namibia is included as a shaded extra).

The total potential DALYs that TB could inflict in South Africa are between 10,070,106.53 and 18,250,705.05 depending on whether Namibia was considered; however, up to 94.5% of this was averted in 2004 due to the current intervention mix. Importantly, this includes not only direct healthcare such as curative and preventive medicine, but also includes broader interventions such as sanitation and access to health, as well as confounders such as strain subtype and genetic susceptibility to the disease. As a result of these factors, South Africa’s actual burden of TB was 1,009,837.3 DALYs. Of this, 86.6 DALYs (0.009%) were unavertable with existing interventions (too small to be seen at the scale of Fig. 5).

### Subdividing existing DALYs

The weighted CPP for South Africa was US$ 404.45, with the lowest weighted CPP being achieved by Yemen, at a cost of US$ 14.29. The Disease Control Priorities Project (DCPP) reports a lowest theoretical cost per patient based on their model of US$ 12 [10], thus, the DALY weighted figure in Yemen is within range of their estimates. When adjusted for purchasing power parity, South Africa had a value of International (Int.)$ 621.33, and Namibia replaced Yemen as the most effective with a DALY weighted value of Int.$ 22.81 (compared to Yemen with a value of Int.$ 24.29). Namibia has managed to continuously improve its health delivery in response to TB since 2004, the year in which it had its highest case notification rate of 16,156. In 2008, this had been reduced to 13,737, with an estimated 83% treatment success rate [19]. By using 2004 burden of disease data with 2008 financing data, it is likely that the figure for Namibia is an underestimation of its true cost-per-patient. Unfortunately, no chronologically matched data exists, which is a limitation of this model, and so it is the best estimate available. Furthermore, Namibia’s Int.$ was similar to other countries with the lowest purchasing power adjusted DALY weighted cost (Fig. 4), which strengthens the validity of the figure. Hence, it was used to calculate potential improvements from efficiency. The ratio between South Africa and Namibia’s Int.$ values was 0.035, which suggested all but 3.5% of the current burden of TB could be averted with improvements in efficiency.

The cost-efficiency data from the DCPP assumed a baseline efficacy using the treatment active non-infectious intervention of 33% for sub-Saharan Africa, with a best estimate of 71% efficacy. Adopting the treatment latent infection intervention, which is currently not used in sub-Saharan Africa and hence has a baseline efficacy of 0%, would result in a best estimate of 80% efficacy. This means that efficacy could be improved by 2.42-fold (80/33) using existing but non-cost-effective interventions.

**Fig. 6:**
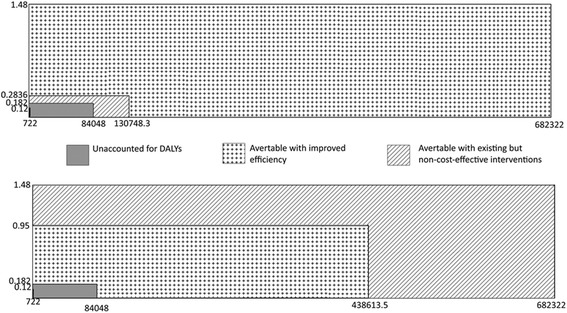
Subdivision of existing DALYs due to TB for South Africa. In the first graph, reductions from improved efficiency are calculated first, followed by reductions through use of existing but non-cost-effective interventions. In the second graph, these reductions are applied first, followed by the reductions from improved efficiency

Using these two ratios (the Int.$ CPP ratio and the DCPP ratio), the existing DALYs from TB were subdivided as illustrated in Fig. 6. If improvements in efficiency were first adopted, then 972,757.1 (96.5%) DALYs could have been averted. The resulting 37,080.24 (3.5%) DALYs could thus be reduced by 2.42-fold with adoption of non-cost-effective interventions to leave a remainder of 15,322.41 DALYs (Fig. 6). Alternatively, if existing non-cost-effective interventions were first adopted, then 592,549.59 (58.68%) DALYs could be averted. Of the remaining 417,288.45 DALYs, 96.33% could be averted with gains in efficiency, again leaving a remainder of 15,322.41 DALYs.

### Limitations

Of the 15,322.41 DALYs remaining (1.52% of the original DALYs for South Africa), 86.6 are accounted for by the unavertable with existing interventions estimate. The remaining 15,235.81 DALYs (1.51% of total DALYs for South Africa) represent the uncertainty in the model, as under ideal conditions all DALYs should be accounted for by the four subsections of the model. It is unclear what this error is attributable to; however, the validity of the model can be confirmed by comparing it to the results of other studies. According to the DCPP, 71% of TB disease burden should currently be averted in South Africa, yet the amended model figures show 89.97% (or 94.5% if Namibia is included) is already being averted. This discrepancy suggests a 1.27-fold difference between the amended models calculations and the DCPP calculations. It is not immediately clear which figure is more accurate, as the DCPP has limitations such as being modelled on Kenya and using deaths as opposed to DALYs to quantify efficacy. However, if the efficacy of the DCPP data was increased 1.27-fold, it would cover the majority of the unaccounted for DALYs. There is also a degree of uncertainty from the WHO sources of data used in the model proposed in this paper, as much of the country-specific data is based on estimates and crude modelling, and the financial data is supplied by the member states and not objectively verified. Finally, there is also a chronological discrepancy between these two data sources, as previously mentioned.

There are also some limitations with the assumptions of the model. The simplified addition of prevalence and incidence to get an estimate of disease burden is crude and requires more refinement. The model can be recalculated on the assumption that either prevalent cases or incident cases are the only contributors to DALY estimates (Additional file 2). However, the WHO uses both prevalence and incidence figures in construction of its DALYs [8], and so it is inaccurate to attribute the total DALYs to one disease burden measure. The WHO uses a Generic Disease Modelling System algorithm to compute prevalence and incidence contributions to DALY estimates, and this algorithm could also potentially be used to construct disease burden estimates. In terms of the accuracy of the DALY pp figures, untreated TB can have a ten year case fatality rate of 70%. Furthermore, those who survive can still be disabled by TB for as long as 10 years and TB is mostly a disease of young to middle-aged adults [20]. As such, the worst case DALY pp estimate of 13.47 DALYs is definitely within the range of what could be expected from TB. In terms of that which is unavertable, a 2006 Tanzanian study suggested that up to 93% of all disease burden was avertable with existing interventions [21]. The results herein suggest that 98.5% of the TB burden is avertable with existing interventions, which is a reasonable agreement with the Tanzanian figure.

The amended model also assumes no relatedness between disease burden and disease severity, and hence chooses the best and worst DALYs pp and disease burdens independently of each other. In reality, it may be impossible to impact the disease burden without impacting the DALYs pp. For example, it is known that TB interventions have different efficacies depending on whether the disease is endemic or rare [10], and hence it may be impossible to achieve both the lowest DALYs pp and the lowest disease burden simultaneously, as the model assumes. The assumptions that disease burden and DALY data can be generalised from one country to another also brings in uncertainty, as there is a high likelihood of confounding variables playing a role. However, the identification and elucidation of these confounders, which lead to such low DALY pp or disease burden scores, are themselves health systems questions worthy of research.

This model also does not fully account for the host of potential confounding variables, such as economies of scope (health systems gains may benefit multiple disease outcomes) and climate. While these are acknowledged as limitations of the model, no attempt is made to control or adjust the model for them.

Furthermore, even if the model is assumed to be generally valid, there are still limitations with its applicability. Firstly, the amended model only looks at research area and not at all issues important in priority setting such as, for example, equity. It is therefore important that this model be viewed as a component to feed into a more complex framework, such as the Combined Approach Matrix model, and not an end to itself. In addition, while the model suggests the burden of disease which could be reduced by research area, it does not quantify how much should be spent on each of those research areas, as different research incurs different costs and has varying levels of cost-effectiveness [22]. If this data were available, then the areas of the model could be weighted as such to reflect cost-effectiveness. However, given that basic research is typically more expensive than health systems research, it would most likely exaggerate the results of the amended model. Thus, research into the cost-effectiveness of research is required, although this is a requirement for all priority setting processes, and not just this specific model. Finally, defining what area research falls into is sometimes ambiguous. For example, a TB vaccine could both reduce the unavertable component of the disease, be cheaper than current interventions and be more effectively delivered to the population. As such, a separate ranking method is required when evaluating research proposals on a case by case basis to determine what impact the research is likely to have. Once the various research proposals have been defined, the amended model nonetheless highlights how much of each research area should be invested in. For example, the importance of health systems research investments is clearly highlighted in TB, traditionally a neglected area in research [23], and suggests that any TB research should have health systems at the forefront, even if it is a cross-disciplinary endeavour.

A final limitation to the model is the potential for a disease-specific lens being used to define research priorities, as this would exclude pure health systems research. This could be addressed by summing all the subsections of the amended WHO model across all diseases analysed to get a general idea of how much health systems research is required relative to basic research and biomedical research; however, the validity of such a simple summation is unclear.

## Conclusion

This paper provides the first results at attempting to develop a quantitative-only model and methodology for the ranking of research priorities. At its current stage, it is a grossly simplified model, but is widely applicable with limited data inputs. Further refinements are still required – the inclusion and weighting of relevant confounding variables such as socioeconomic factors and comorbidities could help strengthen the specificity of the model – nor does the model currently take into account the ranking of other relevant criteria such as equity, which would need stakeholder input. Nevertheless, policymakers can use this model in its current form as an efficient way to graphically illustrate burden of disease data for multiple diseases, as well as highlight the relative challenges in tackling those diseases (health systems, basic research, etc.), both within the disease and between diseases. This is useful in steering the desired composition of health research as a whole and health research within a specific disease field.

## Additional files

Additional file 1:
**Country figures (data summary table).** (DOC 351 kb) Additional file 2:
**Disease burden estimates.** (DOC 41 kb)

## Abbreviations

COHRED: Council On Health Research for Development
CPP: Cost per patient
DALY: Disability Adjusted Life Year
DALY pp: DALYs per person
DCPP: Disease Control Priorities Project
Int.$: International dollars
TB: Tuberculosis
WHO: World Health Organization
