# Supplementary material for: Setting priorities in health research using the model proposed by the World Health Organization: development of a quantitative methodology using tuberculosis in South Africa as a worked example
Source: Health Res Policy Syst. 2016 Feb 9;14:10. doi: 10.1186/s12961-016-0081-8 (PMC4746905; doi:10.1186/s12961-016-0081-8)
Supplement: Additional file 1: — Country figures (data summary table). (DOC 351 kb) [file 12961_2016_81_MOESM1_ESM.doc]

Additional File 1: Country figures (Data Summary Table)

| **Country** | **DALYS / 100 000 (2004)^1^** | **Prevalence / 100 000 (2004)^1^** | **Incidence / 100 000 (2004)^1^** | **Disease Burden (column 2+3)** | **DALYS pp (column 1/6)** | **Country Population (2004)^1^** | **Cost Per Patient Treated in local currency (2008 & 2009)^2^** | **DALY weighted cost per patient** | **PPP Adjusted Cost** | **DALY Weighted Cost PPP Adjusted** |
| --- | --- | --- | --- | --- | --- | --- | --- | --- | --- | --- |
| Afghanistan | 1 589 | 372 | 189 | 561 | 2.83 | 18553819 | 333.9346 | 945.8165483 | 689.0832 | 1951.718 |
| Albania | 59 | 27 | 20 | 47 | 1.25 | 3179442 |  | 0 | #DIV/0! | #DIV/0! |
| Algeria | 64 | 130 | 87 | 217 | 0.29 | 27751086 |  | 0 | #DIV/0! | #DIV/0! |
| American Samoa |  | 18 | 11 | 29 | - | 51885 |  | #DIV/0! | #DIV/0! | #DIV/0! |
| Andorra | 11 | 17 | 13 | 30 | 0.35 | 63111 |  | 0 | #DIV/0! | #DIV/0! |
| Angola | 875 | 310 | 270 | 580 | 1.51 | 11742960 |  | 0 | #DIV/0! | #DIV/0! |
| Anguilla |  | 55 | 22 | 77 | - | 9549 |  | #DIV/0! | #DIV/0! | #DIV/0! |
| Antigua and Barbuda | 3 | 5.5 | 4.3 | 9.8 | 0.29 | 66895 |  | 0 | #DIV/0! | #DIV/0! |
| Argentina | 43 | 47 | 35 | 82 | 0.52 | 34420352 | 77.3195 | 40.23454819 | 181.1911 | 94.28594 |
| Armenia | 246 | 109 | 75 | 184 | 1.34 | 3290540 | 108.2037 | 144.7868436 | 157.8949 | 211.2784 |
| Aruba |  | 8.2 | 6.4 | 14.6 | - | 76762 |  | #DIV/0! | #DIV/0! | #DIV/0! |
| Australia | 2 | 7.5 | 5.9 | 13.4 | 0.15 | 17921818 |  | 0 | #DIV/0! | #DIV/0! |
| Austria | 7 | 17 | 13 | 30 | 0.22 | 7894229 |  | 0 | #DIV/0! | #DIV/0! |
| Azerbaijan | 248 | 945 | 416 | 1361 | 0.18 | 7669735 |  | 0 | #DIV/0! | #DIV/0! |
| Bahamas | 91 | 16 | 18 | 34 | 2.67 | 275426 |  | 0 | #DIV/0! | #DIV/0! |
| Bahrain | 59 | 53 | 43 | 96 | 0.62 | 545329 |  | 0 | #DIV/0! | #DIV/0! |
| Bangladesh | 1 362 | 454 | 225 | 679 | 2.01 | 115059015 | 25.1185 | 50.36855858 | 4304.934 | 8632.415 |
| Barbados | 4 | 5.4 | 4.7 | 10.1 | 0.35 | 262637 |  | 0 | #DIV/0! | #DIV/0! |
| Belarus | 223 | 106 | 73 | 179 | 1.24 | 10293127 |  | 0 | #DIV/0! | #DIV/0! |
| Belgium | 8 | 15 | 12 | 27 | 0.29 | 10057529 |  | 0 | #DIV/0! | #DIV/0! |
| Belize | 161 | 43 | 40 | 83 | 1.94 | 214153 |  | 0 | #DIV/0! | #DIV/0! |
| Benin | 458 | 110 | 76 | 186 | 2.46 | 5473217 |  | 0 | 434.9904 | 1070.2 |
| Bermuda |  | 6.8 | 5.3 | 12.1 | - | 61104 |  | #DIV/0! | #DIV/0! | #DIV/0! |
| Bhutan | 588 | 548 | 307 | 855 | 0.69 | 523417 | 3348.1092 | 2304.340641 | 7272.902 | 5005.585 |
| Bolivia (Plurinational State of) | 841 | 243 | 163 | 406 | 2.07 | 7304230 |  | 0 | 300.9444 | 623.5328 |
| Bonaire, Saint Eustatius and Saba | | 6 | 0.69 | 6.69 | - | 18488 |  | #DIV/0! |  | #DIV/0! |
| Bosnia and Herzegovina | 86 | 59 | 53 | 112 | 0.76 | 3462032 |  | 0 | #DIV/0! | #DIV/0! |
| Botswana | 1 063 | 591 | 773 | 1364 | 0.78 | 1546414 | 341.0421 | 265.7866553 | 560.7541 | 437.0163 |
| Brazil | 148 | 62 | 53 | 115 | 1.29 | 159398558 | 573.5483 | 738.2083593 | 656.0267 | 844.3655 |
| British Virgin Islands | | 4.7 | 3.6 | 8.3 | - | 18070 |  | #DIV/0! |  | #DIV/0! |
| Brunei Darussalam | 122 | 85 | 66 | 151 | 0.81 | 282000 |  | 0 | #DIV/0! | #DIV/0! |
| Bulgaria | 51 | 81 | 56 | 137 | 0.37 | 8449971 | 4128.15 | 1543.853009 | 6796.483 | 2541.761 |
| Burkina Faso | 1 581 | 101 | 64 | 165 | 9.58 | 10401025 | 180.0903 | 1725.285272 | 324.6236 | 3109.93 |
| Burundi | 2 333 | 285 | 212 | 497 | 4.69 | 6017127 | 107.0442 | 502.525781 | 228.7816 | 1074.03 |
| CÃ´te d'Ivoire | 2 069 | 387 | 287 | 674 | 3.07 | 14253952 | 222.3401 | 682.527225 | 303.546 | 931.8086 |
| Cambodia | 2 266 | 1318 | 526 | 1844 | 1.23 | 10862069 | 141.3513 | 173.7064967 | 274.932 | 337.8637 |
| Cameroon | 604 | 394 | 317 | 711 | 0.85 | 13589699 | 82.4731 | 70.11554136 | 123.7221 | 105.1839 |
| Canada | 3 | 6.9 | 5.5 | 12.4 | 0.22 | 29009032 |  | 0 | #DIV/0! | #DIV/0! |
| Cape Verde | 1 305 | 270 | 155 | 425 | 3.07 | 385101 | 466.5605 | 1432.266093 | 413.5754 | 1269.61 |
| Cayman Islands |  | 1.9 | 1.5 | 3.4 | - | 31437 |  | #DIV/0! | #DIV/0! | #DIV/0! |
| Central African Republic | 1 708 | 939 | 763 | 1702 | 1.00 | 3247456 | 184.2757 | 184.9444185 | 251.4566 | 252.3691 |
| Chad | 1 931 | 212 | 151 | 363 | 5.32 | 6785842 |  | 0 | #DIV/0! | #DIV/0! |
| Chile | 42 | 32 | 22 | 54 | 0.77 | 14174890 |  | 0 | #DIV/0! | #DIV/0! |
| China | 275 | 146 | 95 | 241 | 1.14 | 1201522570 | 189.2202 | 215.9534553 | 314.8542 | 359.3372 |
| China, Hong Kong SAR | | 125 | 98 | 223 | - | 6048024 |  | #DIV/0! |  | #DIV/0! |
| China, Macao SAR |  | 113 | 88 | 201 | - | 391906 |  | #DIV/0! | #DIV/0! | #DIV/0! |
| Colombia | 153 | 56 | 39 | 95 | 1.61 | 35797965 | 866.0785 | 1397.463438 | 1526.205 | 2462.612 |
| Comoros | 235 | 68 | 36 | 104 | 2.26 | 482401 |  | 0 | #DIV/0! | #DIV/0! |
| Congo | 1 655 | 512 | 430 | 942 | 1.76 | 2658948 | 145.6943 | 256.0136007 | 163.1492 | 286.6853 |
| Cook Islands | 130 | 7.8 | 6.1 | 13.9 | 9.36 | 18252 |  | 0 | #DIV/0! | #DIV/0! |
| Costa Rica | 28 | 41 | 25 | 66 | 0.43 | 3384498 |  | 0 | #DIV/0! | #DIV/0! |
| Croatia | 45 | 39 | 31 | 70 | 0.65 | 4658613 |  | 0 | #DIV/0! | #DIV/0! |
| Cuba | 6 | 14 | 9.6 | 23.6 | 0.27 | 10847842 |  | 0 | #DIV/0! | #DIV/0! |
| CuraÃ§ao |  |  |  | 0 | #DIV/0! | 145939 |  | #DIV/0! | #DIV/0! | #DIV/0! |
| Cyprus | 8 | 4.6 | 3.6 | 8.2 | 1.00 | 837205 |  | 0 | #DIV/0! | #DIV/0! |
| Czech Republic | 8 | 15 | 12 | 27 | 0.29 | 10322561 |  | 0 | #DIV/0! | #DIV/0! |
| Democratic People's Republic of Korea | 260 | 605 | 344 | 949 | 0.27 | 21471407 |  | 0 | #DIV/0! | #DIV/0! |
| Democratic Republic of the Congo | 1 898 | 514 | 327 | 841 | 2.26 | 42650247 | 190.6451 | 430.2699879 | 239.5707 | 540.691 |
| Denmark | 6 | 10 | 8.3 | 18.3 | 0.31 | 5212071 |  | 0 | #DIV/0! | #DIV/0! |
| Djibouti | 3 181 | 871 | 619 | 1490 | 2.13 | 613219 | 20.5778 | 43.93110657 | 30.75249 | 65.65284 |
| Dominica | 12 | 20 | 14 | 34 |  | 71350 |  | #DIV/0! | #DIV/0! | #DIV/0! |
| Dominican Republic | 334 | 116 | 85 | 201 | 1.66 | 7774932 | 357.2499 | 592.7850616 | 522.2096 | 866.5028 |
| Ecuador | 612 | 149 | 88 | 237 | 2.58 | 11173647 | 2419.3625 | 6246.83095 | 4652.62 | 12013.14 |
| Egypt | 75 | 34 | 22 | 56 | 1.34 | 61032081 | 540.3559 | 725.3474818 | 1144.283 | 1536.03 |
| El Salvador | 189 | 43 | 32 | 75 | 2.53 | 5663820 | 905.4206 | 2286.226251 | 1561.07 | 3941.769 |
| Equatorial Guinea | 1 141 | 313 | 206 | 519 | 2.20 | 428005 |  | 0 | #DIV/0! | #DIV/0! |
| Eritrea | 1 705 | 185 | 126 | 311 | 5.48 | 3186063 | 5296.0846 | 29029.17743 | 12103.09 | 66340.09 |
| Estonia | 136 | 57 | 46 | 103 | 1.32 | 1467016 |  | 0 | #DIV/0! | #DIV/0! |
| Ethiopia | 1 976 | 350 | 359 | 709 | 2.79 | 55281054 | 147.4628 | 411.0578664 | 297.7255 | 829.9206 |
| Fiji | 66 | 82 | 42 | 124 | 0.53 | 765725 |  | 0 | #DIV/0! | #DIV/0! |
| Finland | 7 | 10 | 7.8 | 17.8 | 0.38 | 5086368 |  | 0 | #DIV/0! | #DIV/0! |
| France | 8 | 14 | 11 | 25 | 0.32 | 57628904 |  | 0 | #DIV/0! | #DIV/0! |
| French Polynesia |  | 34 | 26 | 60 | - | 211583 |  | #DIV/0! | #DIV/0! | #DIV/0! |
| Gabon | 1 078 | 757 | 597 | 1354 | 0.80 | 1055707 | 262.4412 | 209.0094655 | 267.8666 | 213.3303 |
| Gambia | 1 076 | 378 | 244 | 622 | 1.73 | 1094974 |  | 0 | #DIV/0! | #DIV/0! |
| Georgia | 266 | 354 | 193 | 547 | 0.49 | 5157814 | 1509.878 | 733.8208697 | 2386.581 | 1159.91 |
| Germany | 5 | 10 | 8.2 | 18.2 | 0.28 | 81495194 |  | 0 | #DIV/0! | #DIV/0! |
| Ghana | 1 189 | 173 | 125 | 298 | 3.99 | 16554855 | 480.9518 | 1919.075928 | 912.15 | 3639.627 |
| Greece | 8 | 7.5 | 5.9 | 13.4 | 0.63 | 10574134 |  | 0 | #DIV/0! | #DIV/0! |
| Greenland |  | 227 | 178 | 405 | - | 55631 |  | #DIV/0! | #DIV/0! | #DIV/0! |
| Grenada | 2 | 8.6 | 4.3 | 12.9 | 0.17 | 99313 |  | 0 | #DIV/0! | #DIV/0! |
| Guam |  | 42 | 32 | 74 | - | 143492 |  | #DIV/0! | #DIV/0! | #DIV/0! |
| Guatemala | 361 | 118 | 65 | 183 | 1.97 | 9788284 | 319.6153 | 631.109185 | 464.6974 | 917.5869 |
| Guinea | 1 310 | 329 | 216 | 545 | 2.40 | 7249558 |  | 0 | #DIV/0! | #DIV/0! |
| Guinea-Bissau | 952 | 286 | 207 | 493 | 1.93 | 1103003 | 87.1826 | 168.4353608 | 128.5097 | 248.2786 |
| Guyana | 350 | 124 | 115 | 239 | 1.46 | 726552 | 2169.6615 | 3175.521418 | 3471.152 | 5080.386 |
| Haiti | 1 470 | 393 | 277 | 670 | 2.19 | 7724690 | 18.161 | 39.85560667 | 28.60248 | 62.77018 |
| Honduras | 298 | 133 | 96 | 229 | 1.30 | 5440691 | 487.3005 | 634.7016747 | 837.4317 | 1090.742 |
| Hungary | 41 | 31 | 24 | 55 | 0.74 | 10342427 |  | 0 | #DIV/0! | #DIV/0! |
| Iceland | 4 | 4.4 | 3.7 | 8.1 | 0.50 | 264909 |  | 0 | #DIV/0! | #DIV/0! |
| India | 726 | 383 | 212 | 595 | 1.22 | 946373316 | 44.5227 | 54.31456733 | 111.4199 | 135.9245 |
| Indonesia | 1 167 | 370 | 201 | 571 | 2.04 | 196488446 | 196.7636 | 402.1400916 | 379.7913 | 776.2071 |
| Iran (Islamic Republic of) | 84 | 32 | 21 | 53 | 1.59 | 58808655 | 59.4036 | 94.63038897 | 126.8188 | 202.0233 |
| Iraq | 314 | 68 | 49 | 117 | 2.68 | 19633844 |  | 0 | #DIV/0! | #DIV/0! |
| Ireland | 8 | 14 | 11 | 25 | 0.33 | 3586685 |  | 0 | #DIV/0! | #DIV/0! |
| Israel | 7 | 9.6 | 7.7 | 17.3 | 0.43 | 5164345 |  | 0 | #DIV/0! | #DIV/0! |
| Italy | 5 | 9.5 | 7.7 | 17.2 | 0.27 | 56965197 |  | 0 | #DIV/0! | #DIV/0! |
| Jamaica | 20 | 8.4 | 6.5 | 14.9 | 1.34 | 2439337 | 134.205 | 179.9076925 | 170.1972 | 228.1568 |
| Japan | 16 | 34 | 27 | 61 | 0.27 | 124097649 |  | 0 | #DIV/0! | #DIV/0! |
| Jordan | 15 | 9.2 | 7.2 | 16.4 | 0.91 | 4216536 | 3363.6363 | 3070.542797 | 4183.891 | 3819.324 |
| Kazakhstan | 452 | 366 | 247 | 613 | 0.74 | 16120117 |  | 0 | #DIV/0! | #DIV/0! |
| Kenya | 2 577 | 334 | 357 | 691 | 3.73 | 26642887 | 172.346 | 642.7311529 | 250.6111 | 934.6057 |
| Kiribati | 141 | 689 | 448 | 1137 | 0.12 | 76188 | 275.6205 | 34.1676224 | 488.7805 | 60.59225 |
| Kuwait | 41 | 38 | 30 | 68 | 0.61 | 1687552 |  | 0 | #DIV/0! | #DIV/0! |
| Kyrgyzstan | 481 | 386 | 226 | 612 | 0.79 | 4542747 |  | 0 | #DIV/0! | #DIV/0! |
| Lao People's Democratic Republic | 778 | 782 | 281 | 1063 | 0.73 | 4677981 | 358.091 | 262.2097581 | 769.3386 | 563.3431 |
| Latvia | 149 | 113 | 84 | 197 | 0.76 | 2528893 |  | 0 | #DIV/0! | #DIV/0! |
| Lebanon | 29 | 14 | 12 | 26 | 1.10 | 3364891 | 840 | 921.9041237 | 1143.778 | 1255.302 |
| Lesotho | 958 | 415 | 643 | 1058 | 0.91 | 1761558 | 193.75 | 175.5114521 | 381.1072 | 345.2319 |
| Liberia | 1 684 | 443 | 261 | 704 | 2.39 | 2039922 | 87.5674 | 209.4212289 | 168.1252 | 402.0787 |
| Libya | 42 | 53 | 40 | 93 | 0.45 | 4687106 |  | 0 | #DIV/0! | #DIV/0! |
| Lithuania | 157 | 98 | 77 | 175 | 0.90 | 3652438 |  | 0 | #DIV/0! | #DIV/0! |
| Luxembourg | 6 | 12 | 9.4 | 21.4 | 0.28 | 401804 |  | 0 | #DIV/0! | #DIV/0! |
| Madagascar | 1 248 | 512 | 268 | 780 | 1.60 | 12728117 | 152.4593 | 243.9281038 | 275.5454 | 440.8604 |
| Malawi | 1 563 | 284 | 378 | 662 | 2.36 | 9772164 | 153.3289 | 362.0917645 | 316.1826 | 746.6767 |
| Malaysia | 423 | 125 | 87 | 212 | 2.00 | 20205446 | 29.0697 | 58.0252983 | 45.23463 | 90.29171 |
| Maldives | 141 | 80 | 56 | 136 | 1.03 | 243256 | 787.9856 | 814.7232279 | 916.0959 | 947.1805 |
| Mali | 2 123 | 107 | 71 | 178 | 11.93 | 9564231 | 205.7761 | 2454.534107 | 311.9557 | 3721.063 |
| Malta | 3 | 7.2 | 5.8 | 13 | 0.20 | 383314 |  | 0 | #DIV/0! | #DIV/0! |
| Marshall Islands | 206 | 598 | 340 | 938 | 0.22 | 50565 |  | 0 | #DIV/0! | #DIV/0! |
| Mauritania | 1 783 | 563 | 300 | 863 | 2.07 | 2228453 | 149.1446 | 308.1547906 | 254.7963 | 526.4468 |
| Mauritius | 53 | 43 | 23 | 66 | 0.80 | 1122040 |  | 0 | #DIV/0! | #DIV/0! |
| Mexico | 59 | 35 | 23 | 58 | 1.01 | 90691331 | 91.5204 | 92.45519387 | 123.5525 | 124.8145 |
| Micronesia (Federated States of) | 250 | 476 | 247 | 723 | 0.35 | 105825 |  | 0 | #DIV/0! | #DIV/0! |
| Monaco | 4 | 1 | 0.78 | 1.78 | 2.36 | 32574 |  | 0 | #DIV/0! | #DIV/0! |
| Mongolia | 534 | 331 | 228 | 559 | 0.96 | 2288370 | 310.0646 | 296.2766851 | 575.7542 | 550.1516 |
| Montenegro |  | 41 | 34 | 75 | - | 10707 |  | #DIV/0! | #DIV/0! | #DIV/0! |
| Montserrat |  | 4.7 | 10 | 14.7 | - | 26523600 |  | #DIV/0! | #DIV/0! | #DIV/0! |
| Morocco | 213 | 131 | 103 | 234 | 0.91 | 15409381 | 27.9314 | 25.45905405 | 38.50031 | 35.09246 |
| Mozambique | 1 872 | 490 | 520 | 1010 | 1.85 | 41552659 | 321.3893 | 595.6103604 | 544.1064 | 1008.358 |
| Myanmar | 487 | 677 | 406 | 1083 | 0.45 | 1603865 | 102.7053 | 46.16912925 | 249.3781 | 112.103 |
| Namibia | 1 048 | 1622 | 1720 | 3342 | 0.31 | 9863 | 53.3333 | 16.72111618 | 72.76991 | 22.8149 |
| Nauru | 66 | 94 | 74 | 168 | 0.39 | 21064150 |  | 0 | #DIV/0! | #DIV/0! |
| Nepal | 686 | 236 | 163 | 399 | 1.72 | 30485798 | 37.6492 | 64.75775557 | 80.81318 | 139.0011 |
| Netherlands | 5 | 11 | 8.6 | 19.6 | 0.25 | 15320112 |  | 0 | #DIV/0! | #DIV/0! |
| New Caledonia |  | 34 | 26 | 60 | - | 186358 |  | #DIV/0! | #DIV/0! | #DIV/0! |
| New Zealand | 7 | 13 | 9.9 | 22.9 | 0.31 | 3623421 |  | 0 | #DIV/0! | #DIV/0! |
| Nicaragua | 234 | 83 | 56 | 139 | 1.68 | 4535802 | 65.8333 | 110.9159269 | 132.5836 | 223.3768 |
| Niger | 1 124 | 272 | 150 | 422 | 2.66 | 8871631 |  | 0 | #DIV/0! | #DIV/0! |
| Nigeria | 1 847 | 300 | 180 | 480 | 3.85 | 107452627 | 242.1602 | 932.0279386 | 314.4452 | 1210.239 |
| Niue | 57 | 48 | 38 | 86 | 0.66 | 2204 |  | 0 | #DIV/0! | #DIV/0! |
| Northern Mariana Islands | | 108 | 84 | 192 | - | 54852 |  | #DIV/0! |  | #DIV/0! |
| Norway | 4 | 8.9 | 7 | 15.9 | 0.24 | 4333931 |  | 0 | #DIV/0! | #DIV/0! |
| Oman | 31 | 17 | 14 | 31 | 1.02 | 2182619 |  | 0 | #DIV/0! | #DIV/0! |
| Pakistan | 1 118 | 488 | 231 | 719 | 1.55 | 124121817 | 132.1347 | 205.4233881 | 355.7163 | 553.0148 |
| Palau | 162 | 56 | 34 | 90 | 1.80 | 16804 |  | 0 | #DIV/0! | #DIV/0! |
| Panama | 75 | 50 | 47 | 97 | 0.78 | 2622903 | 746.3093 | 579.0816264 | 1166.108 | 904.8148 |
| Papua New Guinea | 1 318 | 544 | 355 | 899 | 1.47 | 4595463 | 199.8906 | 293.1322737 | 307.9001 | 451.5243 |
| Paraguay | 304 | 69 | 49 | 118 | 2.58 | 4685320 | 162.0019 | 417.6702821 | 278.9296 | 719.1311 |
| Peru | 439 | 195 | 148 | 343 | 1.28 | 23404523 |  | 0 | #DIV/0! | #DIV/0! |
| Philippines | 1 429 | 662 | 306 | 968 | 1.48 | 67703053 | 74.057 | 109.3082958 | 127.7074 | 188.4964 |
| Poland | 33 | 34 | 26 | 60 | 0.56 | 38364540 |  | 0 | #DIV/0! | #DIV/0! |
| Portugal | 40 | 43 | 40 | 83 | 0.48 | 10077548 |  | 0 | #DIV/0! | #DIV/0! |
| Puerto Rico |  | 3.4 | 3.4 | 6.8 | - | 3668794 |  | #DIV/0! | #DIV/0! | #DIV/0! |
| Qatar | 153 | 60 | 47 | 107 | 1.43 | 495126 |  | 0 | #DIV/0! | #DIV/0! |
| Republic of Korea | 111 | 170 | 86 | 256 | 0.43 | 44365820 |  | 0 | #DIV/0! | #DIV/0! |
| Republic of Moldova | 437 | 264 | 174 | 438 | 1.00 | 4361061 | 1009.7572 | 1007.459276 | 1665.615 | 1661.825 |
| Romania | 201 | 205 | 154 | 359 | 0.56 | 22810195 | 582.5 | 326.5404906 | 754.5337 | 422.9799 |
| Russian Federation | 593 | 216 | 139 | 355 | 1.67 | 148866314 | 4202.8952 | 7015.587313 | 6931.291 | 11569.9 |
| Rwanda | 2 613 | 230 | 176 | 406 | 6.44 | 5648306 | 140.7065 | 905.7282048 | 262.8331 | 1691.858 |
| Saint Kitts and Nevis | 42 | 3.6 | 2.8 | 6.4 | 6.55 | 42511 |  | 0 | #DIV/0! | #DIV/0! |
| Saint Lucia | 17 | 13 | 10 | 23 | 0.74 | 145255 |  | 0 | #DIV/0! | #DIV/0! |
| Saint Vincent and the Grenadines | 56 | 41 | 25 | 66 | 0.85 | 108089 |  | 0 | #DIV/0! | #DIV/0! |
| Samoa | 97 | 24 | 18 | 42 | 2.31 | 166662 |  | 0 | #DIV/0! | #DIV/0! |
| San Marino | 2 | 2.6 | 2 | 4.6 | 0.37 | 25406 |  | 0 | #DIV/0! | #DIV/0! |
| Sao Tome and Principe | 1 308 | 123 | 106 | 229 | 5.71 | 125488 | 269.6106 | 1539.696806 | 350.919 | 2004.034 |
| Saudi Arabia | 143 | 22 | 17 | 39 | 3.66 | 18117969 |  | 0 | #DIV/0! | #DIV/0! |
| Senegal | 1 584 | 225 | 144 | 369 | 4.29 | 8143140 | 240.7377 | 1033.532861 | 348.5668 | 1496.464 |
| Serbia | 46 | 41 | 34 | 75 | 0.62 | 10753700 | 2140.5652 | 1317.263506 | 3400.898 | 2092.849 |
| Seychelles | 34 | 58 | 34 | 92 | 0.37 | 74016 |  | 0 | #DIV/0! | #DIV/0! |
| Sierra Leone | 2 863 | 946 | 479 | 1425 | 2.01 | 3915066 |  | 0 | #DIV/0! | #DIV/0! |
| Singapore | 36 | 48 | 39 | 87 | 0.41 | 3385486 |  | 0 | #DIV/0! | #DIV/0! |
| Sint Maarten (Dutch part) | |  |  | 0 | #DIV/0! |  |  | #DIV/0! |  | #DIV/0! |
| Slovakia | 17 | 21 | 17 | 38 | 0.44 | 5353767 |  | 0 | #DIV/0! | #DIV/0! |
| Slovenia | 11 | 20 | 15 | 35 | 0.32 | 1959752 |  | 0 | #DIV/0! | #DIV/0! |
| Solomon Islands | 688 | 274 | 150 | 424 | 1.62 | 346599 | 234.4359 | 380.483345 | 378.8794 | 614.9114 |
| Somalia | 1 441 | 494 | 285 | 779 | 1.85 | 6484228 | 34.0004 | 62.90615422 | #DIV/0! | #DIV/0! |
| South Africa | 2 484 | 785 | 898 | 1683 | 1.48 | 40542036 | 274.0589 | 404.455542 | 421.0137 | 621.3311 |
| South Sudan |  | 267 | 146 | 413 | - |  |  | #DIV/0! | #DIV/0! | #DIV/0! |
| Spain | 13 | 23 | 19 | 42 | 0.31 | 39318577 |  | 0 | #DIV/0! | #DIV/0! |
| Sri Lanka | 236 | 102 | 66 | 168 | 1.41 | 18089720 | 727.932 | 1024.110769 | 1390.038 | 1955.612 |
| Sudan | 1 576 | 225 | 135 | 360 | 4.38 | 29367076 | 223.1417 | 976.7135992 | 316.62 | 1385.877 |
| Suriname | 58 | 107 | 67 | 174 | 0.34 | 430102 |  | 0 | #DIV/0! | #DIV/0! |
| Swaziland | 2 216 | 672 | 1120 | 1792 | 1.24 | 944404 | 58.4776 | 72.30700612 | 104.6655 | 129.4179 |
| Sweden | 4 | 7 | 5.5 | 12.5 | 0.29 | 8789976 |  | 0 | #DIV/0! | #DIV/0! |
| Switzerland | 3 | 9.9 | 7.9 | 17.8 | 0.17 | 6961228 |  | 0 | #DIV/0! | #DIV/0! |
| Syrian Arab Republic | 142 | 34 | 28 | 62 | 2.28 | 13809349 |  | 0 | #DIV/0! | #DIV/0! |
| Tajikistan | 996 | 408 | 208 | 616 | 1.62 | 5691845 |  | 0 | #DIV/0! | #DIV/0! |
| Thailand | 479 | 248 | 164 | 412 | 1.16 | 59126690 | 822.8734 | 956.3890893 | 1536.614 | 1785.938 |
| The Former Yugoslav Republic of Macedonia | 65 | 36 | 32 | 68 | 0.95 | 1953874 |  | 0 | #DIV/0! | #DIV/0! |
| Timor-Leste | 2 449 | 741 | 498 | 1239 | 1.98 |  | 46.5503 | 92.01133848 | 83.12554 | 164.306 |
| Togo | 2 476 | 117 | 81 | 198 | 12.50 | 3982804 | 338.1159 | 4227.361362 | 527.2362 | 6591.876 |
| Tokelau |  | 1.3 | 0.53 | 1.83 | - | 1524 |  | #DIV/0! | #DIV/0! | #DIV/0! |
| Tonga | 87 | 34 | 24 | 58 | 1.50 | 95691 | 3109.3333 | 4667.955035 | 4004.317 | 6011.569 |
| Trinidad and Tobago | 39 | 17 | 15 | 32 | 1.22 | 1253000 |  | 0 | #DIV/0! | #DIV/0! |
| Tunisia | 72 | 29 | 23 | 52 | 1.39 | 8808669 | 391.2 | 541.9648791 | 657.9767 | 911.5549 |
| Turkey | 108 | 30 | 31 | 61 | 1.76 | 57911273 | 2545.6496 | 4488.371197 | 3106.4 | 5477.06 |
| Turkmenistan | 296 | 367 | 189 | 556 | 0.53 | 4095512 |  | 0 | #DIV/0! | #DIV/0! |
| Turks and Caicos Islands | | 27 | 21 | 48 | - | 14644 |  | #DIV/0! |  | #DIV/0! |
| Tuvalu | 119 | 511 | 303 | 814 | 0.15 | 9188 | 2800 | 409.2320548 | #DIV/0! | #DIV/0! |
| Uganda | 2 213 | 338 | 326 | 664 | 3.33 | 20193432 | 210.5723 | 701.7294892 | 405.0557 | 1349.843 |
| Ukraine | 502 | 163 | 119 | 282 | 1.78 | 51377434 |  | 0 | #DIV/0! | #DIV/0! |
| United Arab Emirates | 77 | 3.7 | 2 | 5.7 | 13.47 | 2232980 |  | 0 | #DIV/0! | #DIV/0! |
| United Kingdom of Great Britain and Northern Ireland | 9 | 16 | 13 | 29 | 0.31 | 57838596 |  | 0 | #DIV/0! | #DIV/0! |
| United Republic of Tanzania | 1 606 | 233 | 225 | 458 | 3.51 | 29064223 |  | 0 | #DIV/0! | #DIV/0! |
| United States of America | 3 | 6.5 | 5.6 | 12.1 | 0.22 | 263468980 |  | 0 | #DIV/0! | #DIV/0! |
| Uruguay | 342 | 28 | 23 | 51 | 6.70 | 3200342 |  | 0 | #DIV/0! | #DIV/0! |
| US Virgin Islands |  | 9.9 | 7.7 | 17.6 | - | 106260 |  | #DIV/0! | #DIV/0! | #DIV/0! |
| Uzbekistan | 449 | 522 | 255 | 777 | 0.58 | 22466866 | 968.4528 | 559.6581339 | 3391.529 | 1959.927 |
| Vanuatu | 372 | 138 | 86 | 224 | 1.66 | 164254 | 646.874 | 1074.324977 | 582.5191 | 967.4447 |
| Venezuela (Bolivarian Republic of) | 61 | 46 | 34 | 80 | 0.76 | 21569680 | 122.0891 | 92.88576327 | 209.7002 | 159.5406 |
| Viet Nam | 533 | 335 | 205 | 540 | 0.99 | 72769366 | 72.1895 | 71.30576741 | 155.372 | 153.47 |
| Wallis and Futuna Islands | | 93 | 72 | 165 | - | 14077 |  | #DIV/0! |  | #DIV/0! |
| West Bank and Gaza Strip | | 7.3 | 3.6 | 10.9 | - | 2479672 |  | #DIV/0! |  | #DIV/0! |
| Yemen | 363 | 145 | 87 | 232 | 1.57 | 14530275 | 9.1323 | 14.2931169 | 15.5204 | 24.29124 |
| Zambia | 1 972 | 427 | 591 | 1018 | 1.94 | 8692599 | 164.5421 | 318.8069679 | 165.9756 | 321.5844 |
| Zimbabwe | 1 911 | 409 | 807 | 1216 | 1.57 | 11469872 | 171.2814 | 269.1761782 | #DIV/0! | #DIV/0! |

1- Mathers C, Boerma T, Ma Fat D: The Global Burden of Disease 2004. 2004.

2- World Health Organisation: Global Tuberculosis Control - Surveillance, Planning, Financing. 2004.
