# Supplementary material for: Setting priorities in health research using the model proposed by the World Health Organization: development of a quantitative methodology using tuberculosis in South Africa as a worked example
Source: Health Res Policy Syst. 2016 Feb 9;14:10. doi: 10.1186/s12961-016-0081-8 (PMC4746905; doi:10.1186/s12961-016-0081-8)
Supplement: Additional file 2: — Disease burden estimates. (DOC 41 kb) [file 12961_2016_81_MOESM2_ESM.doc]

Additional File 2: Disease Burden Estimates

| **Data Type** | Prevalence Only | | Incidence Only | | Prevalence + Incidence | |
| --- | --- | --- | --- | --- | --- | --- |
|  | **Value** | **Country** | **Value** | **Country** | **Value** | **Country** |
| **DALY Data** |  |  |  |  |  |  |
| DALYs PP | 3.16 | SA | 2.77 | SA | 1.48 | SA |
| Best DALYs PP | 0.20 | Kiribati | 0.31 | Kiribati | 0.12 | Kiribati |
| Worst DALYs PP | 21.16 | Togo | 38.38 | UAE | 13.47 | UAE |
| **Disease Burden Data** |  |  |  |  |  |  |
| DB / 100000 | 785.00 | SA | 898.00 | SA | 1683.00 | SA |
| Disease Burden | 318254.98 |  | 364067.48 |  | 682322.47 |  |
| Best DB /100000 | 1.00 | Monaco | 0.53 | Tokelau | 1.78 | Monaco |
| Best DB | 405.42 |  | 214.87 |  | 721.65 |  |
| Worst DB/100000 | 1622.00 | Namibia | 1720.00 | Namibia | 3342.00 | Namibia |
| Worst DB | 657591.82 |  | 697323.02 |  | 1354914.84 |  |
| 2nd Worst DB/10000 | 1318.00 | Cambodia | 1120.00 | Swaziland | 1844.00 | Cambodia |
| 2nd Worst DB | 534344.03 |  | 454070.80 |  | 747595.14 |  |
